# Predicting effective drug combinations using gradient tree boosting based on features extracted from drug-protein heterogeneous network

**DOI:** 10.1186/s12859-019-3288-1

**Published:** 2019-12-09

**Authors:** Hui Liu, Wenhao Zhang, Lixia Nie, Xiancheng Ding, Judong Luo, Ling Zou

**Affiliations:** 1 Lab of Information Management, Changzhou University, Jiangsu, China; 2 School of Information Science and Engineering, Changzhou University, Jiangsu, China; 3 Information Center, Changzhou University, Jiangsu, 213164 China; 4 Department of Radiation Oncology, the Affiliated Changzhou No.2 People’s Hospital of Nanjing Medical University, Changzhou, China

**Keywords:** Drug combination, Random walk, Heterogenous network

## Abstract

**Background:**

Although targeted drugs have contributed to impressive advances in the treatment of cancer patients, their clinical benefits on tumor therapies are greatly limited due to intrinsic and acquired resistance of cancer cells against such drugs. Drug combinations synergistically interfere with protein networks to inhibit the activity level of carcinogenic genes more effectively, and therefore play an increasingly important role in the treatment of complex disease.

**Results:**

In this paper, we combined the drug similarity network, protein similarity network and known drug-protein associations into a drug-protein heterogenous network. Next, we ran random walk with restart (RWR) on the heterogenous network using the combinatorial drug targets as the initial probability, and obtained the converged probability distribution as the feature vector of each drug combination. Taking these feature vectors as input, we trained a gradient tree boosting (GTB) classifier to predict new drug combinations. We conducted performance evaluation on the widely used drug combination data set derived from the DCDB database. The experimental results show that our method outperforms seven typical classifiers and traditional boosting algorithms.

**Conclusions:**

The heterogeneous network-derived features introduced in our method are more informative and enriching compared to the primary ontology features, which results in better performance. In addition, from the perspective of network pharmacology, our method effectively exploits the topological attributes and interactions of drug targets in the overall biological network, which proves to be a systematic and reliable approach for drug discovery.

## Background

Traditional “one drug, one target” treatment can not always lead to desirable therapeutic effect on complex diseases, because biological pathways and networks are often redundant and robust to single point perturbations [1]. Drug combination perturbs the biological network through synergistic and synthetic lethal effects, and inhibits more effectively the activity level of pathogenic genes [2, 3]. Previous studies have shown that combination drugs can effectively inhibit cancer cell growth or promote cancer cell apoptosis, with reduced toxicity and side effects than single target drugs [4]. Even more promising, drug resistance can be decreased or even overcome through combination therapy [5]. Therefore, therapeutic schemes from single- to multi-target drugs play an increasingly important role in the treatment of complex diseases [6].

Despite the increasing successes of combination drugs in inhibiting cancer cell proliferation, most of them are discovered by clinical experience or by occasional chances [5, 6]. Developing combinations of targeted agents is more difficult than developing a single agent [7], as inhibiting the cross-talks among multiple pathways depends on our insight into the pathway interdependencies underlying the cancer cell proliferation and survival in a specific cancer type [8, 9]. The high-throughput screening (HTS) experiments currently used to evaluate drug combinations are still time- and cost- consuming because they rely heavily on the search for a large number of possible target combinations [10–12]. So, there is an urgent demand for rational and systematically in silico methods to narrow down the candidates for combinatorial drugs for wet-lab experimental validations [13].

Quite a few computational methods have been proposed to predict cancer sensitivity to combinatorial drugs [1, 4, 14–16]. The existing methods can be roughly divided into two categories: system biology-based methods [17] and network-based analysis [15, 18]. System biology-based methods mathematically model the perturbation of drugs using biochemical reactions and kinetic parameters, which are often limited to small scale and well-studied signaling pathway. Network-based methods often exploit genomic, chemical and pharmacological properties to build an overall network composed of the associations among drugs, proteins and pathways, and then adopt scoring rules [19, 20], optimal combination searches [1, 16, 21], machine learning [4, 18] to predict potential drug combinations. As network-based methods integrate various kinds of ontological features and interactions between different subject of interest, some of these methods achieve remarkable performance in predicting drug combinations. For example, Ligeti et al. [20] proposed so-called Target Overlap Score (TOS) prioritization function, which is defined for two drugs as the number of jointly perturbed targets divided by the number of all targets potentially affected by these two drugs, to rank candidate drug combinations. Pang et al. [1] proposed mixed integer linear programming to find balanced target set cover (BTSC) and minimum off target set cover (MOTSC) for combination therapy. Huang et al. [18] propose DrugComboRanker, which first builds a drug functional network based on their genomic profiles, and disease-specific signaling networks based on patients genomic profiles and interactome data, and then prioritize synergistic drug combinations by searching drugs whose targets are enriched in the complementary signaling modules of the disease signaling network. Matlock et al. [21] tried to find drug combinations maximizing sensitivity over tumor cell models while minimizing toxicity over normal cell models, and then proposed a lexicographic search algorithm to find optimal target set. In addition, some methods exploit the concept of synthetic lethality to discover combinatorial drugs [3, 22, 23]. However, most of previous methods are usually limited to the ability to dissect potential molecular mechanisms, or to associate multiple drugs to one disease in huge pharmacological space.

There have been many approaches that integrate multiple heterogeneous networks to infer the associations between biological entities, including lncRNA functions [24–26], lncRNA-disease associations [27], drug-disease associations [28, 29] and gene functions inference [30]. Inspired by heterogeneous network-based inference, we ran random walk with restart on the drug-protein heterogenous network to extract features for drug combinations, and then trained gradient tree boosting classifier using the extracted features to predict new drug combinations. Concretely, we integrated a variety of data sources, including chemical structures of the drugs, protein sequences, and known drug-protein associations, to construct a drug-protein heterogeneous network. The random walk with restart procedure was implemented on the heterogenous network using the combinatorial drug and their targets as the initial probability, respectively. The converged probability distribution was used as feature vector of the drug combination. Based on the probability distribution vectors, we subsequently trained the gradient tree boosting (GTB) classifier, which achieved the AUC of 0.949 by 10-fold cross-validation. We also compared our method to other seven typical classifiers, including kNN, SVM, Logistic regression, Naive Bayes, AdaBoost, Random Forest and LogistBoost. The performance comparison results demonstrate that our proposed model significantly outperformed other traditional methods. From the perspective of network pharmacology, our method effectively make use of the topological attributes and functional interactions of drug targets in the protein-protein network.

## Results

### Drug combination dataset

The set of effective drug combinations was obtained from DCDB 2.0 [31], a typical drug combination database focused on collecting verified drug combinations to facilitate further exploration, including theoretical modeling and simulation of such beneficial drug combinations. In total, the current version(2.0) of DCDB includes 1363 drug combinations (330 approved and 1033 investigational, including 237 unsuccessful usages), covering 904 individual drugs and 805 targets. We selected those combinations that are approved or under trials in DCDB as positive samples. Note that the number of non-effective drug combinations is actually enormous, much larger than that of effective in real world. Therefore, we generated a number of negative samples of drug combinations by randomly picking up pairwise drugs to balance the positive and negative samples in our benchmark set. The strategy of generation of negative samples has been widely adopted in the prediction of drug-target interactions and drug-disease associations [28, 32]. Importantly, the drug set that we selected pairwise combinations is expanded from the individual drugs in DCDB to their most associated 3 drugs according to STITCH, yielding 3266 drugs in total. Finally, the benchmark drug combination set contains 1359 positive combinations and 1359 negative combinations.

### Performance measures

We conducted performance evaluation using 10-fold cross validations. In particular, the training set were randomly divided into ten subsets and each subset had roughly equal size to others. Each subset was in turn used as the test set, and the remaining nine subsets were used as training set. This validation process was repeated ten times and each performance measure was averaged over the ten folds for performance evaluation. A couple of performance measures were used in our experiment, including precision (PRE), recall (REC), F-measure, Matthews correlation coefficient (MCC) and the area under the receiver operating characteristic curve (AUC). They are formally defined as below:

1 $$ Precision=\frac{TP}{(TP+FP) }  $$

2 $$ Recall=\frac{TP}{(TP+FN)}  $$

3 $$ F\ -- \ Measure=\frac{(2*Precision*Recall)}{(Precision+Recall)}  $$

4 $$ MCC=\frac{(TP\times TN-FP\times FN)}{\sqrt{(TP+FP)(TP+FN)(TN+FP)(TN+FN)}}  $$

in which *TP* and *TN* represent the numbers of correctly predicted positive and negative samples, *FP* and *FN* represent the numbers of wrong predicted positive and negative samples, respectively. Additionally, the AUC score is computed by varying the cutoff of the predicted scores from the smallest to the greatest value.

### Impact of parameters on performance

To explore the impact of parameter *λ*, which is the probability of random walker jumping to different type of network, We gradually increased its value from 0.1 to 0.9 at interval of 0.1. The aforementioned metrics obtained by 10-fold cross-validation are shown in Table 1, which demonstrate that *λ* has a moderate impact on the prediction performance of our proposed method. In terms of AUC, the values fit approximately a parabola which hit the top 0.944 at *λ* 0.7, which was thus adopted in our subsequent experiments. For other two parameters introduced in random walk with restart, restart probability *α* and tradeoff *η*, we conducted similar tuning to determine their optimal values that achieve the best performance. As shown in Additional file 1: Table S1 and S2, the AUC measure reached the highest value when *α* and tradeoff *η* were equal to 0.2 and 0.9. According to the results, the restart probability *α* has a negligible effect on the AUC. Generally, the restart probability is a heuristical parameter without any theoretical guide or justification when selecting [33]. However, the heterogeneous network is established based on drug-drug similarity, protein-protein similarity and known drug-protein associations, resulting in a heterogeneous network with quantitative weighted edges. From this perspective, since the random walk simulates the influence of drugs in protein network, the convergence state will have a bias on higher weighted nodes. Therefore, the restart probability may have a slight effect on the final distribution. As a result, we set the three parameters *λ*, *α* and *η* to 0.7, 0.2 and 0.9 in the following performance comparison experiments.

**Table 1:** Impact of the parameter *λ* on the performance of GTB classifer

*λ*	Precision	Recall	F-Measure	MCC	AUC
0.1	0.861	0.852	0.856	0.715	0.929
0.2	0.865	0.853	0.858	0.720	0.930
0.3	0.870	0.854	0.861	0.726	0.934
0.4	0.878	0.861	0.869	0.738	0.939
0.5	0.883	0.871	0.877	0.755	0.941
0.6	0.885	0.868	0.875	0.755	0.941
**0.7**	**0.887**	**0.867**	**0.876**	**0.757**	**0.944**
0.8	0.885	0.865	0.875	0.754	0.943
0.9	0.884	0.864	0.874	0.752	0.943

The boldface figures indicate that GTB classifier achieves the best performance at *λ* equal to 0.7

### Performance comparison to typical classifiers

To demonstrate the outstanding performance of our method, we carried out performance evaluation on the benchmark combination set by comparing our method with seven other typical classifiers, including kNN, SVM, Logistic regression, Naive Bayes, Random Forest, Adaboost and LogitBoost. Based on the derived feature distribution vectors, we implemented these competitive classifiers separately using R package [34] so as to conveniently reproduce our work. For Native Bayes, we adopted the R package e1071 [35] and its default setting. Also, logistic regression and SVM are implemented based on the e1071 R package, and logistic regression was run with default settings, while the misclassification penalty coefficient for SVM varied from 10 to 10000 by interval of 500 to achieve best performance. For KNN, R package kknn [36] was used to run the algorithm, in which the parameter k (k =1, 3, 5, 7 and 9) was enumerated to tune its performance. For the distance metric of kNN, we have tried Manhattan distance, Euclidean distance and Chebyshev distance and found that they yield to similar performance, thereby we adopted Chebyshev distance (q=5) in the performance evaluation. The R package randomForest [37] was used to run random forest algorithm and the number of trees varies from 60 to 500 by interval of 20. For boosting methods Adaboost and Logitboost, the R packages Adabag [38] and caTools were used, where the number of training iterations was tuned from 10 to 100 by interval of 5 and 10, respectively. The performance measures of each comparative method, including precision, recall, F1, MCC and AUC, achieved by the fine-tuned parameters, are shown in Table 2. Apparently, our proposed method significantly outperformed other classifiers in terms of almost all performance metrics.

**Table 2:** Comparison of GTB with other typical classifiers on heterogenous network-derived features

Method	Precision	Recall	F-Measure	MCC	AUC
**GTB**	**0.897**	**0.872**	**0.884**	**0.772**	**0.949**
kNN	0.738	0.833	0.783	0.542	0.768
SVM	0.882	0.779	0.840	0.728	0.859
Logistic	0.499	0.527	0.510	0.014	0.520
Naive Bayes	0.504	0.988	0.770	0.086	0.508
Random forest	0.880	0.841	0.862	0.733	0.866
AdaBoost	0.878	0.854	0.863	0.732	0.866
LogitBoost	0.803	0.820	0.811	0.617	0.808

The boldface figures indicate that GTB achieves the best performance compared with other typical classifiers on heterogenous network-derived features

**Fig. 1:**
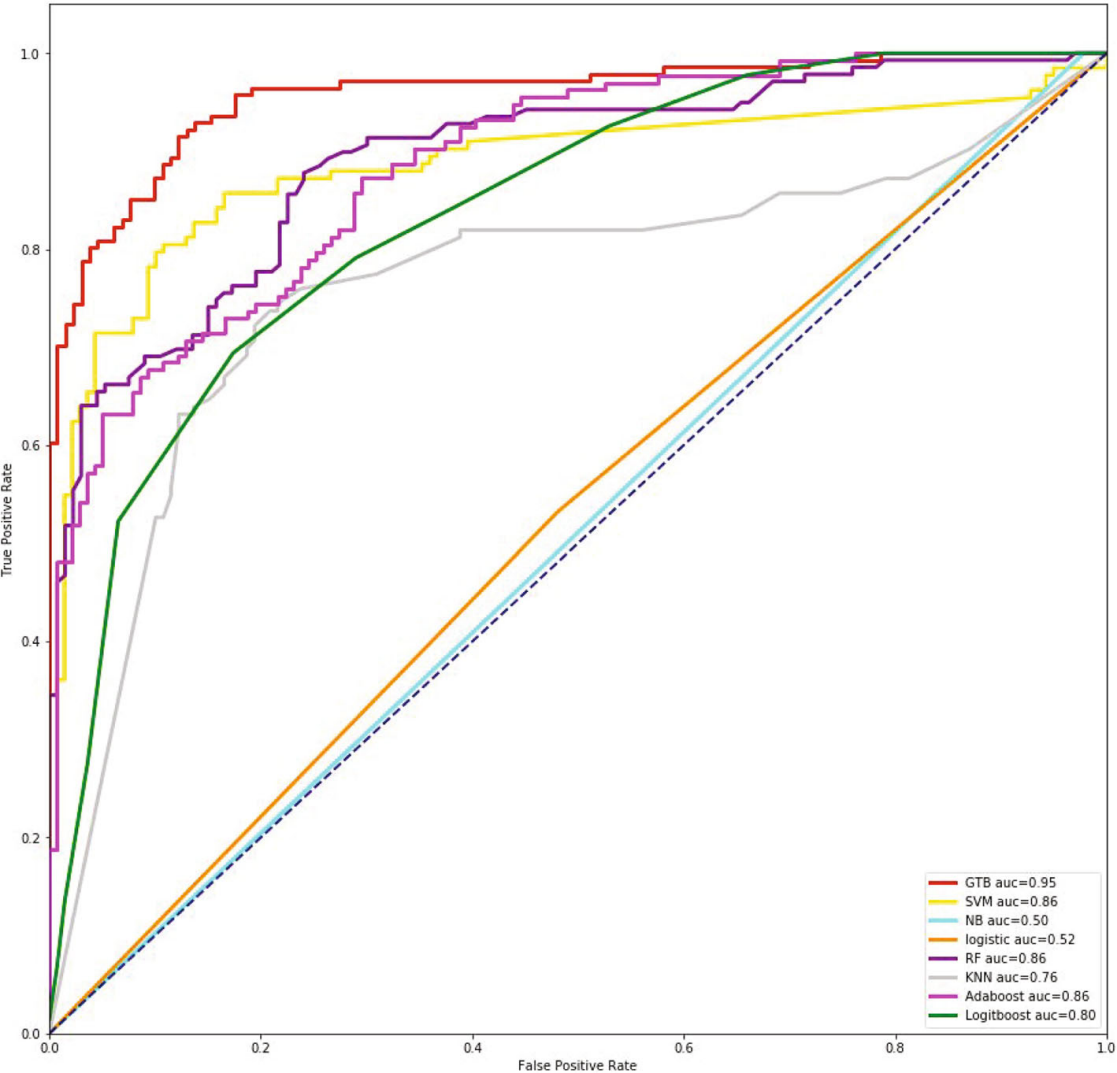
ROC curves of our method and other typical classifiers on benchmark set

To present clear performance comparison, the ROC curves of GTB and other seven classifiers are also illustrated in Fig 1. It can be demonstrated that GTB classifier greatly outperforms all other competitive methods, which achieves the highest AUC value 0.95, followed by Random forest and Adaboost at 0.86. The performance of Naive Bayes is the worst and gets only 0.508 AUC value.

### Performance improvement by heterogenous network-derived features

To validate the effectiveness of the features extracted from drug-protein heterogeneous network, we conducted performance comparison between the primary ontology features and heterogenous network-based features. Due to different number of individual drugs and target proteins involved in drug combinations, we can not directly concatenate the drug fingerprints and protein GO annotations to construct feature vectors that are inconsistent in dimension. Instead, we first unified the chemical fingerprints of individual drugs in a combination, i.e. union of individual fingerprint vectors, as well as the union of GO terms of target proteins of individual drugs. Next, we concatenated the union sets of chemical fingerprints and GO annotations for each pair of drug combinations as the input features of the GTB classifier. The performance measures are shown in Table 3. It can be demonstrated that the performance of GTB classifier with input derived from heterogeneous network-based features is vastly superior to that with primary ontology features. For example, the AUC value increased from 0.528 to 0.949 for GTB classifier. Moreover, we conducted performance comparison for other typical classifiers to validate the advantage of our extracted feature from drug-protein heterogenous network. As shown in Tables 2 and 3, the performance of all these classifiers were greatly boosted by extracting features from the random walk with restart on the heterogenous network.

**Table 3:** Comparison of GTB with other typical classifiers on primary ontology features

Method	Precision	Recall	F-Measure	MCC	AUC
**GTB**	**0.526**	**0.53**	**0.523**	**0.052**	**0.528**
kNN	0.514	0.514	0.513	0.028	0.516
SVM	0.509	0.491	0.478	-0.019	0.491
Logistic	0.506	0.506	0.506	0.012	0.504
Naive Bayes	0.479	0.479	0.478	-0.043	0.46
Random forest	0.499	0.499	0.478	-0.002	0.499
AdaBoost	0.501	0.501	0.425	0.002	0.497
LogitBoost	0.499	0.499	0.479	-0.002	0.495

The boldface figures indicate that GTB achieves the best performance compared with other 7 typical classifiers trained on primary ontology features

## Discussion

In this paper, we proposed a computational method for predicting effective combination drugs based on features derived from drug-protein heterogenous network by random walk with restart. In order to verify our proposed method, we conducted plenty of empirical experiments to compare the performance of our method to other typical classifiers on the benchmark dataset we constructed previously, and the experimental results significantly demonstrated that our method achieves state-of-the-art performance. Note that the input of the GTB classifier is the output of random walk with restart on the heterogeneous network, which is the probability distribution vector only accounting for 6,074 dimensions. Therefore, we believed that the heterogeneous network-derived features are more informative and have been dimension-reduced compared to the high-dimensional primary ontology features that may lead to curse of dimensionality when performing classification. As a result, the performance of GTB and other classifiers are significantly improved. In addition, the majority of current methods to predict drug combinations are limited to their size, in which pairwise drugs are most used. However, our proposed method can expand the size of drug combinations, which appreciably increases the practicality.

It is worth noting that the protein network introduced in the random walk with restart is helpful to dig into the biological mechanism of drug combinations in vivo. In fact, the final probability distribution of certain drug combination derived by random walk with restart strongly suggests the indications of the drug combination to some extent. Taking the pairwise combination Docetaxel and Capecitabine as an example, which has been approved by FDA to treat metastatic breast cancer. We ranked the protein nodes according to the probability distribution, the protein with the highest probability is ENSP00000315644 and the third is ENSP00000252029, which are both encoded by *Tyms* gene. It has been shown that the polymorphisms of *Tyms* gene are associated with etiology of neoplasia, including breast cancer. In addition, the fourth and fifth are ENSP00000269571 and ENSP00000275493, which are encoded by *Erbb2* and *Egfr*, respectively, are all highly linked to breast cancer. To further evaluate the potential of the protein network, we exemplified another pair of drug combination, Atorvastatin and Proguanil, which currently has no official indication. The resulting probability distribution derived by random walk with restart of this drug combination shows that the protein ENSP00000396308 encoded by *Dhfr* has the highest probability value. It has been demonstrated that diseases associated with *Dhfr* include megaloblastic anemia due to dihydrofolate reductase deficiency and megaloblastic anemia. Expectedly, quite a few works have demonstrated the pharmacological effect of Atorvastatin and Proguanil on Anemia. For example, Vahid et al. [39] validated that Atorvastatin can soften human red blood cells, and physical deformation of the red blood cells underlies pathological manifestations of sickle cell anemia and hypercholesterolemia. Another trial demonstrated the effectiveness of Proguanil in treatment of malarial anemia [40]. Therefore, anemia may be a potential indication of the drug combination Atorvastatin and Proguanil. In summary, we draw the conclusion that the probability distribution derived by random walk can effectively reveal the indication of drug combinations.

We further checked the positive samples that are falsely classified, as negative samples are randomly generated. We found that the falsely determined samples by our method have low similarity to other samples. In fact, most existing computational models, which aim at the prediction of drug-target interactions, drug-disease associations, often hold the assumption that similar compounds are likely to interact with similar target proteins and thereby play similar therapeutic efficacy in cellular micro-environment. These computational methods have achieved superior performance, and greatly narrowed down the number of candidate drug targets and reveal new indications of approved drugs. Under this assumption, the prediction accuracy often relies on the close associations of tested samples with known samples that have been validated by wet-lab experiments, such as drug combinations and drug-target interactions. In terms of network medicine, the influence of drug molecule would perturb the cellular network via signal cascade reactions and protein interaction network. Many computational methods have taken into account this consideration, and adopted random walks and diffusion on network to capture the perturbation of the drugs.

However, there are always some samples located far from validated samples in the feature space. For instance, some new drugs have low similarity to other drugs, and some proteins have low similarity to other protein in different protein family. As a result, similarity-based or network diffusion-based computational methods tend to encounter failure in predicting drug combinations or drug-target interactions composed of such drugs or proteins. Fortunately, the emergence of large-scale experimental data derived from high-throughput screening technique can strongly motivate the novelty of methods to predict synergistic drugs or effective drug combinations.

## Conclusion

In this paper, we proposed a gradient tree boosting (GTB) classifier based on heterogeneous network-derived features to predict effective drug combinations. The heterogeneous network integrates the drug similarity network, protein similarity network and known drug-protein associations. Next, we ran random walk with restart (RWR) on the heterogenous network using the combinatorial drugs and their associated targets as the initial probability, and obtained the converged probability distribution as the feature vector of each drug combination. The heterogeneous network-derived features introduced in our method are more informative and enriching compared to the primary ontology features. The GTB classifier trained based on the heterogeneous network-derived features outperforms seven typical classifiers and traditional boosting algorithms. Moreover, our case studies show that our method is helpful in revealing the indications of drug combinations. From the perspective of network pharmacology, our method effectively exploits the topological attributes and interactions of drug targets in the overall biological network, which proves to be a systematic and reliable approach for drug discovery.

## Methods

### Overview of our methodology

**Fig. 2:**
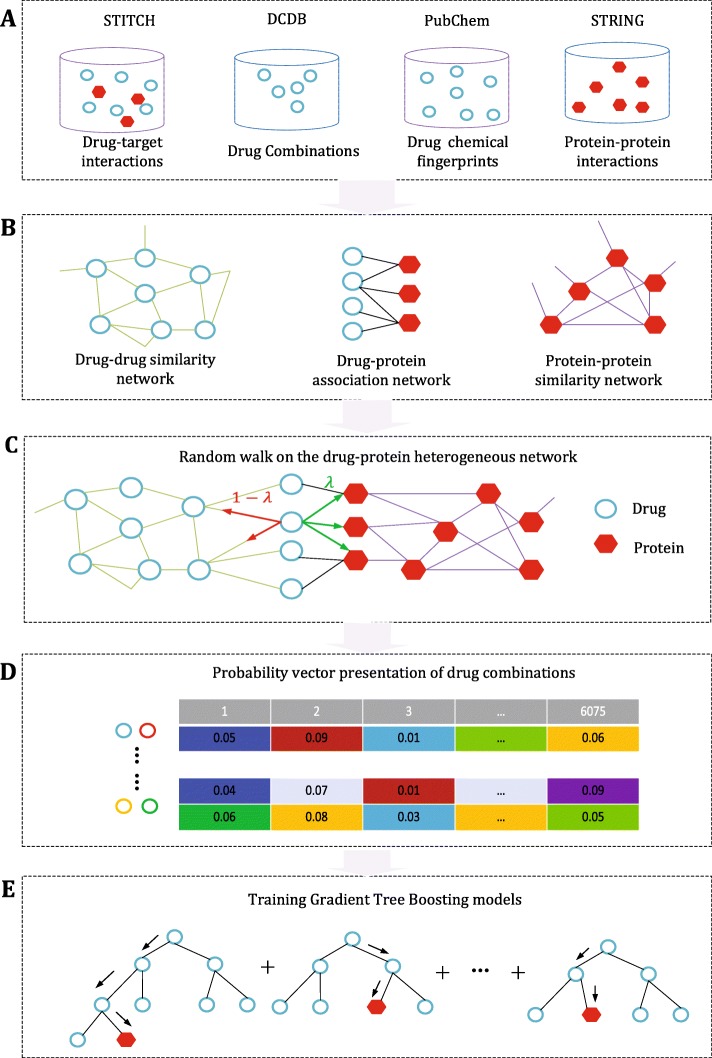
Illustrative diagram of the proposed method. **a** Data collection from drug and protein-related databases; **b** Construction of drug-drug similarity network, protein-protein similarity network and drug-protein association network; **c** Random walk with restart on drug-protein heterogenous network; **d** Feature representations of drug combinations via feature extraction process; **e** Training gradient tree boosting classifier

We first constructed the benchmark drug combination set composed of positive samples derived from public databases and negative samples that were randomly generated. For individual drugs included in the benchmark set, we collected a variety of related characteristics, including chemical fingerprints, drug targets and drug-protein associations, as shown in Fig. 2a. These ontology features of drugs and proteins were used to compute the drug-drug similarities and protein-protein similarities. Together with the known drug-protein associations, we constructed the drug-protein heterogeneous network. Next, the random walk with restart on heterogeneous network proposed in our previous work [28] was conducted for each drug combinations as initial state, as illustrated in Fig. 2b-c. The probability distribution when the random walk reaches steady state was used as the feature vector of the drug combination. Based on the feature representation of the drug combinations, the gradient tree boosting (GTB) classifier was trained to predict new effective drug combinations.

### Drug-protein associations

We selected drug-protein associations from STITCH database [41], which is a comprehensive database that collected compound-protein interactions from different sources: biochemical experiments, external databases, text mining and computational predictions. STITCH has computed a confidence score for each interaction ranging from 0 to 1,000, which indicates the confidence of the compound-protein interaction supported by four types of evidences. We first used a confidence threshold 0.5 (corresponding to 500 combined score in STITCH) to remove low-confidence target proteins, because we think too low-confidence targets are probable unauthentic ones. Next, we selected top 3 from the rest of target proteins of each drug. If one drug has less than 3 target proteins with confidence score higher than 0.5, we then took only those targets into account. In total, we got 210,235 drug-protein associations regarding to 3,266 unique drugs (drug set are built by selecting top 3 similar drugs, see following subsection for details). Formally, denoted by *D*=(*d*_1_,*d*_2_,...,*d*_*n*_) and *P*=(*p*_1_,*p*_2_,...,*p*_*m*_) the drug and protein node set, and *A* the adjacent matrix of drug-protein associations with element *a*_*ij*_ equal to the confidence score if there is validated interaction between drug *i* and protein *j*, and *a*_*ij*_=0 otherwise.

### Drug-drug similarity network

We expanded the list of individual drugs by selecting top 10 most similar drugs to each single agent included in DCDB, according to the chemical-chemical combined scores that were derived from STITCH [41]. After removal of duplicate drugs, 3266 unique drugs were obtained. Similar compounds are likely to interact with similar target proteins and thereby play similar therapeutic efficacy in cellular micro-environment [42], allowing us to find new drug combinations by introducing similar drugs to known ones. Therefore, we believe that the expanded list of drugs can increase the opportunity for discovery of novel drug combinations.

Next, we generate the chemical fingerprint of the drugs to calculate the similarity measurement of each pair of drugs. Similar to our previous work [28], we applied PaDEL software [43] to compute the chemical fingerprints using the SMILES string of a drug, and obtain an 880-d binary vector for each drug. The element 1 of the binary vector represents that the drug contains the corresponding chemical fingerprint, and 0 otherwise. Subsequently, Jaccard score, a widely used similarity measure, is calculated based on the chemical fingerprints as the chemical similarities for pairwise drugs. The Jaccard score is generally defined as the intersection size divided by the union size of two individual sets, which is shown as follows:

5 $$ S^{(d1)}_{ij}=\frac{|\vec{d_{i}}\cap \vec{d_{j}}|}{|\vec{d_{i}}\cup \vec{d_{j}}|}  $$

Further, the bipartite network projection algorithm, a method inspired by the network-based resource-allocation dynamics [44], was adopted to compute another drug similarity measure based on known drug-protein associations. In the drug-protein bipartite network, each drug node equally allocates the original resource to its associated protein nodes, and successively the assigned resource of each protein node is equally transferred back to its neighborhood drugs. As a result, the proportion of the resource of drug *d*_*i*_ conveyed to drug *d*_*j*_ in such allocation process represents the strength of association between two drugs. Suppose the initial resource of each drug node is one-unit, the second drug similarity measure, denoted by $S^{(d2)}_{ij}$, can be formulated as below:

6 $$ S^{(d2)}_{ij}=\frac{1}{k(d_{j})}\sum_{l=1}^{m}\frac{a_{il}a_{jl}}{k(p_{l})}  $$

in which *k*(*d*_*j*_) and *k*(*p*_*l*_) are the degrees of drug *d*_*j*_ and protein *p*_*l*_ in the drug-protein association network. Intuitively, more common associated protein nodes the pairwise drugs share, higher similarity the drugs have. Particularly, if the associated proteins of two drugs are not overlapped, i.e. no common associated protein exists, the similarity is denoted by 0.

Finally, these two aforementioned drug-drug similarities were integrated into a comprehensive measurement using the probability disjunction formula as below:

7 $$ S_{ij}^{(d)}=1-\left(1-S_{ij}^{(d1)}\right)*\left(1- S_{ij}^{(d2)}\right)  $$

### Protein-protein similarity network

Correspondingly, we constructed the protein-protein similarity network based on two different similarity measures, including protein sequence similarity and GO semantic similarity. By using R package biomaRt (2.40.4) [45, 46], the protein sequences can be readily obtained from Ensembl genome database (2018 updated), which is dedicated to curating gene-related information to encourage genome analysis [47]. The sequence similarity $S_{ij}^{(p1)}$ between protein *p*_*i*_ and protein *p*_*j*_ was computed by using the R package Protr (1.6-2) [48], in which the Smith-Waterman algorithm is applicable.

Similar drugs are supposed to interact with proteins that act in similar biological processes or have similar molecular functions or reside in similar compartments [49]. Therefore, the GO semantic similarity $S_{ij}^{(p2)}$ between protein *p*_*i*_ and protein *p*_*j*_ was calculated using R package GOSemSim (2.10.0) [50]. All three types of ontology features are used in the calculation of semantic similarity.

Likewise, the probability disjunction was used to integrate two aforementioned protein-protein similarities, which is formulated as below:

8 $$ S_{ij}^{(p)}=1-\left(1- S_{ij}^{(p1)}\right)*\left(1-S_{ij}^{(p2)}\right)  $$

where $S_{ij}^{(p)}$ is the comprehensively integrated similarity measurement between protein *p*_*i*_ and protein *p*_*j*_.

### Random walk with restart on heterogenous network

The drug-drug similarity network, protein-protein similarity network and drug-protein association network were combined to construct the drug-protein heterogeneous network *G*=(*V*,*E*). The node set *V*={*D*,*P*}, *V* is the union set of the drug and protein nodes. The edge set *E*={*E*_*dd*_∪*E*_*dp*_∪*E*_*pd*_∪*E*_*pp*_}, where *E*_*dd*_, *E*_*pp*_, *E*_*dp*_ and *E*_*pd*_ are the drug-drug edge, protein-protein and drug-protein edge collections, respectively.

**Fig. 3:**
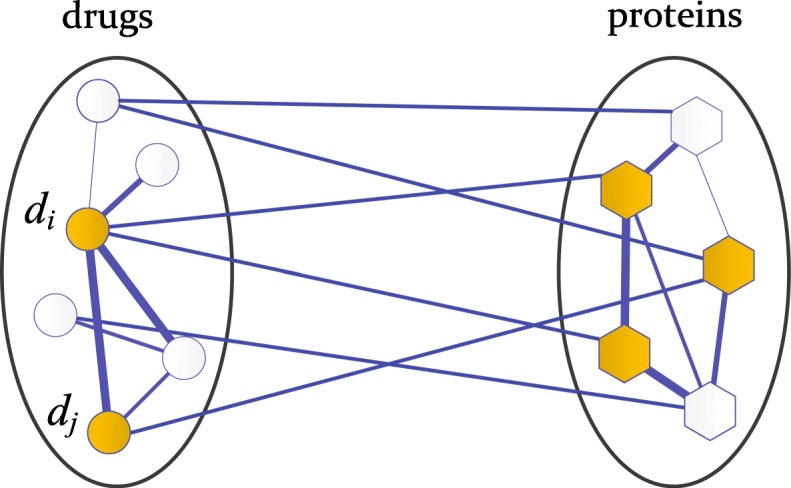
Illustrative diagram of the random walk with restart on drug-protein heterogenous network, starting from two drugs and their targeted proteins

In order to obtain the feature representations of drug combinations, we extended our previous work in which the random walks with restart on the heterogeneous network was developed for single drug repurposing [28]. More precisely, for a drug combination *d*_*i*_ and drug *d*_*j*_, we performed random walk with restart on the heterogeneous network in which these two drugs and their known target proteins act as seed nodes, as shown in Fig. 3. Actually, since the initial probability distribution can be easily extended to more drugs and their targets, the number of individual drugs involved in the combination is not limited to 2 in our method. When the random walk process reaches steady state, the probability distribution vector can be regarded as the perturbation on the protein network by the combinatorial drugs. With the drug-protein heterogeneous network, the transition matrix *T* can be defined as below:

9 $$ T=\left[\begin{array}{cc} T^{(dd)} & T^{(dp)}\\ T^{(pd)}&T^{(pp)} \end{array}\right]  $$

where *T*^(*dd*)^ and *T*^(*pp*)^ are the probability transition matrix from drug nodes (protein) to drug nodes (protein nodes) during the random walk process; *T*^(*dp*)^ denotes the probability transition matrix that drug nodes walk to protein nodes, and *T*^(*pd*)^ denotes the probability transition matrix that protein nodes walk to drug nodes.

Suppose that the random walker starts from a drug node, and then visits one of its targeted proteins with probability *λ*, or visits any other drug nodes with probability (1- *λ*) in the heterogeneous network. If *λ*=0, the random walker can only stay within the networks where it starts. According to the drug-drug similarity, the transition probability from drug *d*_*i*_ to drug *d*_*j*_ can be defined as below:

10 $$  T_{ij}^{(dd)}= \left\{\begin{array}{cc} S_{ij}^{(d)}/\sum_{k=1}^{n}S_{ik}^{(d)},& if\sum_{l=1}^{m}a_{il}=0\\ (1-\lambda) S_{ij}^{(d)}/\sum_{k=1}^{n}S_{ik}^{(d)}, & \text{otherwise}. \end{array}\right.  $$

where *S*_*ij*_ is the similarity between ith drug and jth drug, *a*_*il*_ is the association confidence score between ith drug and lth protein. The sum of *a*_*il*_ equaling to 0 indicates that the drug has no approved or predicted association with any proteins. Similarly, the transition probability from protein *p*_*i*_ to protein *p*_*j*_ can be defined based on the protein-protein similarity as below:

11 $$  T_{ij}^{(pp)}=\left\{\begin{array}{cc} S_{ij}^{(p)}/\sum_{k=1}^{m}S_{ik}^{(p)},& if\sum_{l=1}^{n}a_{li}=0\\ (1-\lambda) S_{ij}^{(p)}/\sum_{k=1}^{m}S_{ik}^{(p)}, & \text{otherwise}. \end{array}\right.  $$

where *S*_*ij*_ is the similarity between ith protein and jth protein, *a*_*li*_ is the association score between lth drug and ith protein.

Accordingly, the transition probability from drug *d*_*i*_ to protein *p*_*j*_ is defined as:

12 $$ T_{ij}^{(dp)}=\left\{\begin{array}{cc} \lambda a_{ij}/\sum_{l=1}^{m}a_{il,} &if\sum_{l=1}^{m}a_{il}\neq 0 \\ 0, & \text{otherwise}. \end{array}\right.  $$

The transition probability from protein *p*_*i*_ to drug *d*_*j*_ is defined as:

13 $$  T_{ij}^{(pd)}=\left\{\begin{array}{cc} \lambda a_{ji}/\sum_{l=1}^{n}a_{li,} &if\sum_{l=1}^{n}a_{li}\neq 0 \\ 0, & \text{otherwise}. \end{array}\right.  $$

Provided that *P*(*t*) is a (*n*+*m*)-dimension probability vector at step *t*, in which *P*(*t*)[i] represents the probability of the random walker visiting node *i*(drug or protein), the random walk process can be iteratively calculated as below:

14 $$ P{(t+1)}=(1-\alpha)T'P{(t)}+\alpha P_{0}  $$

where *α* is the restart probability, and *P*_0_ is the initial probability distribution vector of a set of seed nodes consisting of a combinatorial drugs and their targeted proteins. Take the drug combination *d*_*i*_ and *d*_*j*_ as an example, *d*_*i*_ and *d*_*j*_ are employed as the seed nodes in the drug network and each seed node is given equal probability 1/2. By giving rest drug nodes probability 0, the initial probability matrix with respect to drugs can be constructed. Correspondingly, the protein nodes related to drug *d*_*i*_ and drug *d*_*j*_ are used as seed nodes in protein network and equal probabilities are allocated to these protein nodes so that the sum of the probabilities is 1. As shown in Fig. 3, there are three targeted proteins and thus each protein is given initial probability 1/3. Let $P^{(d)}_{0}$ and $P^{(p)}_{0}$ be the initial probability vectors of drugs and proteins separately, the initial probability *P*_0_ for drug-centric random walk can be defined as follows:

15 $$ P_{0}=\left[\begin{array}{cc} \eta P_{0}^{(d)}\\ (1-\eta) P_{0}^{(p)} \end{array}\right]  $$

where *η*∈[0,1] is a tradeoff parameter to balance the weight of importance between the drug nodes and protein nodes. In our experiments, *η* is set to 0.5. If the difference between twice iteration is lower than 1e-10, the random walk is supposed to reach steady state. Once the random walk process converges, the probability distribution is used as the feature vector of the drug combination.

### Building gradient tree boosting classifier

Based on the feature vectors produced by the random walk on drug-protein heterogenous network for each pair of drug combination, we built a gradient tree boosting (GTB) classification model, referred to as gradient boosting regression or decision tree (GBRT or GBDT). Gradient tree boosting is an efficacious machine learning method that has achieved desirable performance in both classification and regression problems [51–53]. In fact, Caruana and Niculescu-Mizil have conducted comprehensive performance evaluation on eight different binary classification problems by comparing boosted trees algorithm with other nine typical classifiers, including SVMs, Neural Nets, Logistic regression, Naive Bayes, memory-based learning, Random Forests, Decision Trees, Bagged Trees and Boosted Stumps. Their conclusion showed that boosted tree-based algorithm achieved best performance [54]. Another empirical performance evaluation has also demonstrated that boosted decision trees perform exceptionally well when the dimensionality of the input is not too high [55]. Therefore, we adopted the GTB algorithm to build our classification model.

Formally, the decision function of GTB is initialized as:

16 $$ \theta_{0}(x)=arg\,min\sum_{i=1}^{N}L(y_{i},c)  $$

where *N* is the number of drug combinations contained in the training set. The gradient tree boosting algorithm repeatedly constructs *K* different classification subtrees *h*(*x*,*a*_1_), *h*(*x*,*a*_2_),..., *h*(*x*,*a*_*K*_), each of which is separately trained based on a subset of randomly selected samples from the training set, and then iteratively establishes the additive function *θ*_*k*_(*x*):

17 $$ \theta_{k}(x)=\theta_{k-1}(x)+b_{k} h(x,a_{k})  $$

in which *b*_*k*_ and *a*_*k*_ are the weight and parameter vector of the *k*-th classification subtree *h*(*x*,*a*_*k*_). The loss function *L*(*y*,*θ*_*k*_(*x*)) is defined as:

18 $$ L(y,\theta (x))=log(1+exp(-y\theta (x)))  $$

where *y* is a binary value representing the real class of the combination and *θ*(*x*) is the decision function. In order to minimize the loss function *L*(*y*,*θ*_*k*_(*x*)), both *b*_*k*_ and *a*_*k*_ are iteratively optimized by applying grid search. In this paper, grid search strategy was adopted to tune the optimal hyperparameters of GTB by 10-fold cross-validation on the constructed drug combination dataset. Finally, the optimal number of trees of the GTB is 300, and the tuned depth of the trees is 13.

## Supplementary information

**Additional file 1** **Table S1**. Impact of the restart probability *α* on the performance of GTB classifer. **Table S2**. Impact of the tradeoff parameter *η* on the performance of GTB classifer. **Table S3**. Impact of significant parameters involved in GTB classifier. **Table S4-S8**. Parameter tuning for competitive methods.

## Data Availability

The source codes, datasets and additional files used in this work are all available at https://github.com/hliu2016/SynerDrug.

## Abbreviations

GO: Gene ontology
GTB: Gradient tree boosting
kNN: K-NearestNeighbor
RWR: Random walk with restart
SVM: Support vector machine
