# Supplementary material for: Predicting effective drug combinations using gradient tree boosting based on features extracted from drug-protein heterogeneous network
Source: BMC Bioinformatics. 2019 Dec 9;20:645. doi: 10.1186/s12859-019-3288-1 (PMC6902475; doi:10.1186/s12859-019-3288-1)
Supplement: Supplementary file 1 — Additional file 1 Table S1. Impact of the restart probability α on the performance of GTB classifer. Table S2. Impact of the tradeoff parameter η on the performance of GTB classifer. Table S3. Impact of significant parameters involved in GTB classifier. Table S4-S8. Parameter tuning for competitive methods. [file 12859_2019_3288_MOESM1_ESM.pdf]

Supplementary files

November 2019

1 supplementary tables

Table S1: Impact of the restart probability α on the performance of GTB classifier.

α	Precision	Recall	F-measure	MCC	AUC
0.2	0.883	0.871	0.877	0.755	0.941
0.4	0.880	0.848	0.864	0.734	0.937
0.6	0.878	0.852	0.864	0.734	0.941
0.8	0.872	0.848	0.858	0.731	0.938

Table S2: impact of the tradeoff parameter η on the performance of GTB classifier.

η	Precision	Recall	F-measure	MCC	AUC
0.1	0.849	0.831	0.839	0.683	0.920
0.2	0.856	0.860	0.858	0.711	0.930
0.3	0.882	0.858	0.870	0.743	0.939
0.4	0.885	0.861	0.873	0.749	0.941
0.5	0.887	0.867	0.876	0.757	0.945
0.6	0.886	0.864	0.874	0.753	0.945
0.7	0.894	0.871	0.882	0.767	0.945
0.8	0.896	0.872	0.883	0.769	0.948
0.9	0.897	0.872	0.884	0.772	0.949

Table S3: Impact of significant parameters involved in GTB classifier

n-estimators	Precision	Recall	F-measure	MCC	AUC
300	0.890	0.864	0.876	0.758	0.941
350	0.890	0.863	0.876	0.757	0.941
400	0.890	0.864	0.876	0.758	0.941
450	0.890	0.864	0.876	0.758	0.940
500	0.890	0.863	0.876	0.757	0.940
550	0.889	0.864	0.876	0.757	0.940
600	0.889	0.863	0.875	0.755	0.940
650	0.889	0.862	0.875	0.755	0.940
700	0.889	0.862	0.875	0.755	0.940
750	0.888	0.862	0.874	0.754	0.940
800	0.889	0.862	0.875	0.755	0.940
max-depth	Precision	Recall	F-measure	MCC	AUC
6	0.865	0.856	0.860	0.723	0.932
8	0.866	0.858	0.862	0.726	0.938
10	0.875	0.861	0.868	0.738	0.936
12	0.872	0.851	0.861	0.728	0.936
13	0.877	0.844	0.860	0.726	0.940
14	0.875	0.858	0.866	0.735	0.936
16	0.874	0.846	0.860	0.725	0.936
learning rate	Precision	Recall	F-measure	MCC	AUC
0.2	0.883	0.871	0.877	0.755	0.941
0.3	0.881	0.861	0.871	0.744	0.937
0.4	0.864	0.868	0.865	0.730	0.939
0.5	0.838	0.859	0.848	0.691	0.878
0.6	0.799	0.846	0.821	0.631	0.826
0.7	0.752	0.799	0.771	0.537	0.759

Table S4: Parameter tuning for KNN

**KNN**					
K	precision	recall	F1	MCC	AUC
3	0.69526	0.864957	0.770632	0.500551	0.742837
5	0.695414	0.865647	0.771005	0.501403	0.743182
7	0.66025	0.927259	0.771084	0.492059	0.725181
9	0.656424	0.93394	0.770673	0.491322	0.722833
Distance	precision	recall	F1	MCC	AUC
Manhattan	0.675414	0.844567	0.742650	0.480903	0.725682
Euclidean	0.695414	0.865647	0.771005	0.501403	0.743182
Chebyshe(q=3)	0.721	0.851027	0.780432	0.530097	0.760874
Chebyshe(q=4)	0.728414	0.841339	0.780661	0.533513	0.763637
Chebyshe(q=5)	0.732631	0.83691	0.781159	0.536166	0.765461
Chebyshe(q=6)	0.733131	0.834911	0.780528	0.535319	0.765234

Table S5: Parameter tuning for Random Forest

**Random Forest**					
Tree Num	precision	recall	F1	MCC	AUC
60	0.880337	0.839637	0.85918	0.727266	0.863251
80	0.877415	0.837037	0.856283	0.721265	0.860427
100	0.879003	0.840431	0.858971	0.725813	0.862573
120	0.882125	0.839844	0.860215	0.728883	0.864148
140	0.884472	0.841041	0.862088	0.732982	0.866145
160	0.881137	0.838986	0.859379	0.727349	0.863509
180	0.88785	0.837201	0.861619	0.733214	0.865898
200	0.881118	0.834948	0.857142	0.724482	0.861755
220	0.886888	0.842952	0.864206	0.736943	0.86824
240	0.878933	0.833276	0.85523	0.719422	0.859299
260	0.884832	0.834803	0.858842	0.727749	0.863458
280	0.885675	0.83638	0.860137	0.729878	0.86457
300	0.882525	0.838894	0.85995	0.728831	0.864123
320	0.884064	0.83708	0.859687	0.728392	0.863831
340	0.889663	0.842084	0.865101	0.739263	0.869321
360	0.889706	0.837384	0.862671	0.735566	0.867287
380	0.885764	0.837483	0.860835	0.73108	0.865171
400	0.888735	0.838411	0.862617	0.735136	0.867137
420	0.885029	0.838305	0.860848	0.731161	0.865225
440	0.889659	0.837057	0.862295	0.735397	0.867315
460	0.888333	0.835735	0.860966	0.732353	0.865741
480	0.88694	0.838226	0.861788	0.733284	0.866279
500	0.887349	0.8397	0.862746	0.734731	0.866989

Table S6: Parameter tuning for SVM

**SVM**					
c	precision	recall	F1	MCC	AUC
10	0.905743	0.781945	0.84675	0.725237	0.858374
210	0.905743	0.778312	0.845517	0.724475	0.857645
410	0.907429	0.778312	0.846225	0.726124	0.858349
610	0.907499	0.778978	0.846637	0.726769	0.858683
810	0.908381	0.779663	0.847428	0.728204	0.859375
1010	0.906703	0.779663	0.84672	0.726561	0.858673
1210	0.908394	0.779654	0.84742	0.728215	0.85937
1410	0.907499	0.778978	0.846637	0.726769	0.858683
1610	0.906588	0.778312	0.845877	0.725296	0.857997
1810	0.908249	0.778978	0.846961	0.72761	0.859083
2010	0.907338	0.778312	0.846177	0.726158	0.8584
2210	0.906658	0.778978	0.846289	0.72594	0.858331
2410	0.906584	0.778312	0.845865	0.725303	0.857997
2610	0.908178	0.778312	0.846549	0.726966	0.85875
2810	0.906584	0.778312	0.845865	0.725303	0.857997
3010	0.908381	0.779663	0.847428	0.728204	0.859375
3210	0.906588	0.778312	0.845877	0.725296	0.857997
3410	0.906584	0.778312	0.845865	0.725303	0.857997
3610	0.905743	0.778312	0.845517	0.724475	0.857645
3810	0.907338	0.778312	0.846177	0.726158	0.8584
4010	0.908381	0.779663	0.847428	0.728204	0.859375
4210	0.906588	0.778312	0.845877	0.725296	0.857997
4410	0.906755	0.779011	0.846341	0.725948	0.858311
4610	0.90665	0.778978	0.846289	0.725934	0.858328
4810	0.908178	0.778312	0.846549	0.726966	0.85875
5010	0.906655	0.778978	0.846277	0.725947	0.858331
5210	0.906588	0.778312	0.845877	0.725296	0.857997
5410	0.907429	0.778312	0.846225	0.726124	0.858349
5610	0.907429	0.778312	0.846225	0.726124	0.858349
5810	0.907412	0.778978	0.846601	0.726795	0.858734
6010	0.905883	0.779645	0.846338	0.725767	0.858312
6210	0.906658	0.778978	0.846289	0.72594	0.858331
6410	0.908265	0.778312	0.846585	0.726939	0.858699
6610	0.907509	0.779011	0.846653	0.726803	0.858715
6810	0.907429	0.778312	0.846225	0.726124	0.858349
7010	0.906584	0.778312	0.845865	0.725303	0.857997
7210	0.907429	0.778312	0.846225	0.726124	0.858349
7410	0.907429	0.778312	0.846225	0.726124	0.858349
7610	0.908183	0.778312	0.846537	0.726979	0.858753
7810	0.906588	0.778312	0.845877	0.725296	0.857997
8010	0.906588	0.778312	0.845877	0.725296	0.857997
8210	0.907409	0.778978	0.846589	0.726802	0.858734
8410	0.906703	0.779663	0.84672	0.726561	0.858673
8610	0.907424	0.778312	0.846237	0.726111	0.858347
8810	0.906774	0.78033	0.847132	0.727206	0.859006
9010	0.907429	0.778312	0.846225	0.726124	0.858349
9210	0.908265	0.778312	0.846585	0.726939	0.858699
9410	0.907412	0.778978	0.846601	0.726795	0.858734
9610	0.905743	0.778312	0.845517	0.724475	0.857645
9810	0.907496	0.779011	0.846659	0.726773	0.858699

Table S7: Parameter tuning for LogitBoost

**LogitBoost**					
nIter	precision	recall	F1	MCC	AUC
5	0.722204	0.722368	0.719373	0.445081	0.721663
10	0.802566	0.789531	0.793469	0.591569	0.794128
15	0.751433	0.733989	0.741509	0.490749	0.744135
20	0.79773	0.804827	0.800515	0.597528	0.798134
25	0.745207	0.775025	0.758554	0.511151	0.754275
30	0.791091	0.794428	0.792015	0.588161	0.79371
35	0.757278	0.762632	0.758929	0.518059	0.758518
40	0.796649	0.802683	0.799117	0.593772	0.796285
45	0.763947	0.783625	0.77288	0.541569	0.77026
50	0.796171	0.804483	0.799748	0.593436	0.796372
55	0.781941	0.780456	0.780172	0.562078	0.780679
60	0.803458	0.808416	0.805302	0.603255	0.801584
65	0.777474	0.783504	0.779973	0.559652	0.779746
70	0.803111	0.820192	0.810943	0.617113	0.808352
75	0.773724	0.798598	0.785273	0.565455	0.782363
80	0.799281	0.821191	0.8096	0.611602	0.805326
85	0.774445	0.797582	0.785405	0.564571	0.781877
90	0.801372	0.822064	0.811251	0.614073	0.806778
95	0.770648	0.801396	0.785296	0.562493	0.780777
100	0.800013	0.820912	0.809765	0.612427	0.805774

Table S8: Parameter tuning for AdaBoost

**AdaBoost**					
nIter	precision	recall	F1	MCC	AUC
5	0.806963	0.802859	0.804363	0.611474	0.805868
15	0.839297	0.843393	0.840755	0.682227	0.841135
25	0.855255	0.845808	0.849706	0.703949	0.851622
35	0.863191	0.859599	0.860791	0.724326	0.862055
45	0.857132	0.84984	0.85295	0.708447	0.853805
55	0.871861	0.851836	0.861457	0.727648	0.863981
65	0.867665	0.854387	0.860503	0.724253	0.862279
75	0.870735	0.859972	0.865008	0.732736	0.86646
85	0.872824	0.854393	0.863167	0.732062	0.866077
95	0.868813	0.854495	0.861443	0.731843	0.861036
